# Biomechanical Comparison of Osteoporotic Distal Radius Fractures Fixed by Distal Locking Screws with Different Length

**DOI:** 10.1371/journal.pone.0103371

**Published:** 2014-07-31

**Authors:** Xiong Liu, Wei-dong Wu, Ya-feng Fang, Mei-chao Zhang, Wen-hua Huang

**Affiliations:** 1 Department of Anatomy, Southern Medical University, Guangdong Provincial Key Laboratory of Medical Biomechanics, Guangzhou, Guangdong, P.R. China; 2 Department of Orthopaedics, Shilongboai Hospital (The Eight People’s Hospital of Dongguan), Dongguan, Guangdong, P.R. China; 3 Department of Orthopaedics, Wuzhou Red Cross Hospital, Wuzhou, Guangxi, P.R. China; Georgia Regents University, United States of America

## Abstract

**Objectives:**

To evaluate the postoperative stability of osteoporotic distal radius fractures fixed with distal locking screws with different length.

**Methods:**

A comminuted extra-articular dorsally unstable distal radius fracture, treated with volar locking plate system, was created. The 18 specimens were randomized into 3 groups based on distal locked screws with different length: Group A had unicortical screws with 50% length to the dorsal cortex. Group B had unicortical screws with 75% length to the dorsal cortex. Group C had bicortical screws. Axial compression and bending loads were imposed on the models before and after cycling testing as well as load to clinical and catastrophic failure.

**Results:**

Minimum change in stiffness was observed before and after fatigue for all groups. The final stiffness to bending forces was statistically similar in all groups, but stiffness to axial compression was statistically significant different: Group A approached significance with respect to groups B and C (P = 0.017, 0.009), whereas stiffness in group B and C was statistically similar (P = 0.93). Load to clinical failure was significantly less for group A (456.54±78.59 N) compared with groups B (580.24±73.85 N) and C (591.07±38.40 N). Load to catastrophic failure was statistically similar between groups, but mean values for Group A were 18% less than means for Group C.

**Conclusions:**

The volar locking plate system fixed with unicortical locking screws with at least 75% length not only produced early stability for osteoporotic distal radius fractures, but also avoided extensor tendon complications due to dorsal screw protrusion.

## Introduction

Distal radius fracture is a common type of skeletal injuries in clinical orthopedics, accounting for approximately 10% of all fractures. The incidence increases with the development of our aging society with increasing in osteoporosis. It consists of a compressive fracture of the dorsal cortex with comminution and tensile fracture of the volar cortex. Dorsal comminution is the most common fracture pattern observed with distal radius fractures [1], [2], [3], [4], [5], [6], [7], [8]. The majority of cases have traditionally been treated by means of external fixation with plaster cast or splints after manual reduction, which often cannot maintain the biomechanical stability due to the unstable displaced distal radius fractures results in comminution. Thus treatment of open reduction and internal fixation is necessary for fractures with dorsal comminution [9], [10], [11].

Because of the lesser extensor tendon complications and increased stiffness of constructs, the fixations with volar locking plates, instead of dorsal plating have become more common. But the increased use of volar locking plates for distal radius fixation leads to several problems such as flexor tendon synovitis, extensor tenosynocitis, extensor pollicis longus rupture and so on, though only a few authors have reported these complications [12], [13], [14], [15], [16], [17], [18], [19], [20]. These dorsal extensor tendons may be injured by improper surgical technique such as inappropriate plate placement and drill penetration of the dorsal cortex to the overlying tendon during operation, or by dorsally prominent screw tips. The first improper technique can be minimized with extensive care, while the second one is tough because it’s difficult for us to see the prominent screw tips dorsally with remarkable clarity with standard fluoroscopy resulting from the anatomic landmark of Lister’s tubercle. So we wonder whether unicortial fixation with appropriate length can be used for distal radius fractures instead of bicortical fixation. If it can provide the equal fixation strength during early period of postoperation, it makes sense to avoid extensor tendon complications because of dorsal screw prominence. Accordingly, the purpose of this study was to clarify whether unicortical distal locked configurations could maintain postoperative biomechanical stability as for extra-articular osteoporotic distal radius fractures compared with bicortical locked fixation. Our null hypothesis was that there is no significant difference between unicortical screw fixation with appropriate lengths and distal bicortical screw fixation.

## Materials and Methods

### Ethics Statement

Ethical approval was obtained from the Human Research Ethics Committee, Southern Medical University, Guangzhou, China. The subjects gave informed consent. And all consent was written in nature regarding body donation for research.

### Specimen preparation

**Figure 1:**
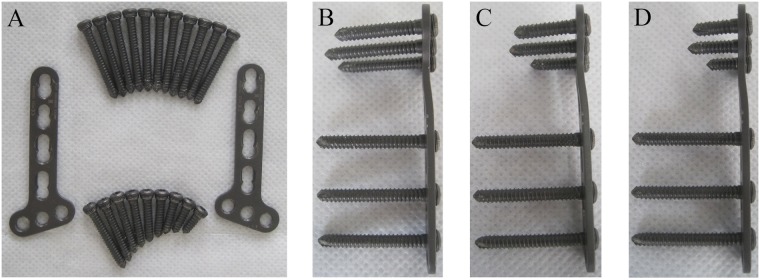
Different fixations in the distal fragment according to groups: (A) screws and plates used in the experiment, (B) fixation with bicortical screws, (C) fixation with 75% unicortical screws, (D) fixation with 50% unicortical screws.

**Figure 2:**
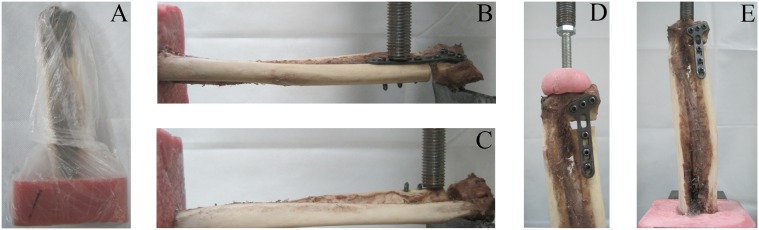
The specimens tested under different loading conditions in a materials testing machine: (A) A specimen was in double plastic wrapping, (B) volar bending, (C) dorsal bending, (D) axial compression, (E) A specimen under axial fatigue test.

18 embalmed cadaveric foreams without osseous defects and any history of upper extremity diseases including injury, arthritis, or tumor were obtained from the Department of Anatomy, Southern Medical University. Bone mineral density data were not available because the bones have been soaked with formalin for many years and may have a tendency to develop similar bone mineral density as well as low bone quality at last with the gradual loss of mineral substance. The specimens were randomized into 3 groups of 6 specimens according to the distal locked screws with different lengths in the distal fragment: Group A had unicortical locking screws with 50% length to the dorsal cortex. Group B had 75% length unicortical locking screws. Group C had bicortical locking screws (Fig. 1). The specimens were dissected of soft tissues maintaining the interosseous membrane and radioulnar ligaments intact. They were stored frozen (–20°C) in double plastic wrapping (Fig. 2A) and completely thawed for 2 hours at room temperature prior to experiment.

The plate is made up of medical titanium alloy (Ti6Al4V). All oblique angulated T-volar locking plates were placed according to the manufacturer’s recommendation with position of the plate as distally as possible without risking penetration of the articular surface and bent manually to conform to the anatomy of the volar distal radius. Plates were temporarily partially affixed to the intact radius and holes were predrilled to assure restoration of alignment postosteotomy. Plates were removed, and a standard 1-cm-wide segment of bone, centered 2 cm proximal to the tip of radial styloid, was performed parallelly with a saw having both dorsal and volar cortex comminution to simulate comminuted extra-articular unstable distal radius fracture [21]. Volar locking plate was fixed with 3.5-mm threaded bicortical and unicortical locking screws with care to ensure that the locking screws were properly engaged into their threaded holes. The most distal diaphyseal hole of every plate in all groups was not filled as a result of the osteotomy cut as seen in actual clinical scenarios. The proximal radial and ulnar diaphyses were potted in acrylic thermoplastic resin in a vertical and neutral position.

### Biomechanical testing

The cadaveric ulna and radius were kept moist with normal saline throughout the experimental procedure. Each specimen was placed in a materials testing machine (ElectroForce 3510, BOSE, USA) and was tested under 3 different conditions of axial compression, dorsal and volar bending. We created a custom-built device attached to the joint surface with acrylic resin powder and liquid to simulate axial load transmission in vivo (Fig. 2D). Cantilever model was applied during bending tests, and mean stiffness of the 3 groups were calculated under different loading conditions.

Each fixation construct was first tested under dorsal and volar bending loads of 50 N at a rate of 5 N/s, and then a static axial compression load was applied at 15 N/s to a maximum of 150 N (Fig. 2). The maximum load applied represents the upper end of estimated physiological forces with wrist motion. Each specimen was subsequently cycled 5000 times in axial compression (sinusoidal waveform, 10–150 N compression at 1 Hz), which was used to simulate physiologic loading over the initial 6-week healing time. For the stability of the whole fatigue process, the load we applied was conducted from a screw shaft attached to the testing machine (Fig. 2E). After cyclic loading, post-testing in the three prior loading conditions was repeated. Finally, destructive testing in axial compression was performed at a rate of 0.5 mm/s to determine load to clinical failure, which was defined as 2 mm of displacement at the osteotomy gap and subsequent catastrophic failure, defined as loosening of the screw-plate interfaces, fractures of distal radius near the location of the implant or deformation and breakage of the implant.

Stiffness values of axial and bending loading were calculated as the slope of the straight-line region of the curve. The loads to clinical and catastrophic failure phenomena were observed and stiffness values were obtained according to characteristic load-displacement curves. Load to catastrophic failure was defined as a turning point in the continuous curve, usually denoted by a sudden drop in the force.

### Statistical analysis

Means and standard deviations were calculated for descriptive purposes. One-way analysis of variance was performed and post hoc multiple comparisons with LSD and Dunnett T3 methods were used to distinguish statistical differences under each loading condition according to condition of homogeneity of variance. Independent-Samples T Test was conducted when two samples were from two independent populations. The 3 groups were compared for precycling and postcycling axial, volar and dorsal bending stiffness, clinical and catastrophic failure stiffness, load to 2-mm displacement and load to catastrophic failure. Statistically significant differences between the 3 groups were considered statistically significant with p value less than 0.05, and statistical analysis was performed with SPSS Statistics v20 software (IBM, Armonk, New York). Post-hoc analysis was carried out to help to explain the results if the study did not find any significant effects.

## Results

**Figure 3:**
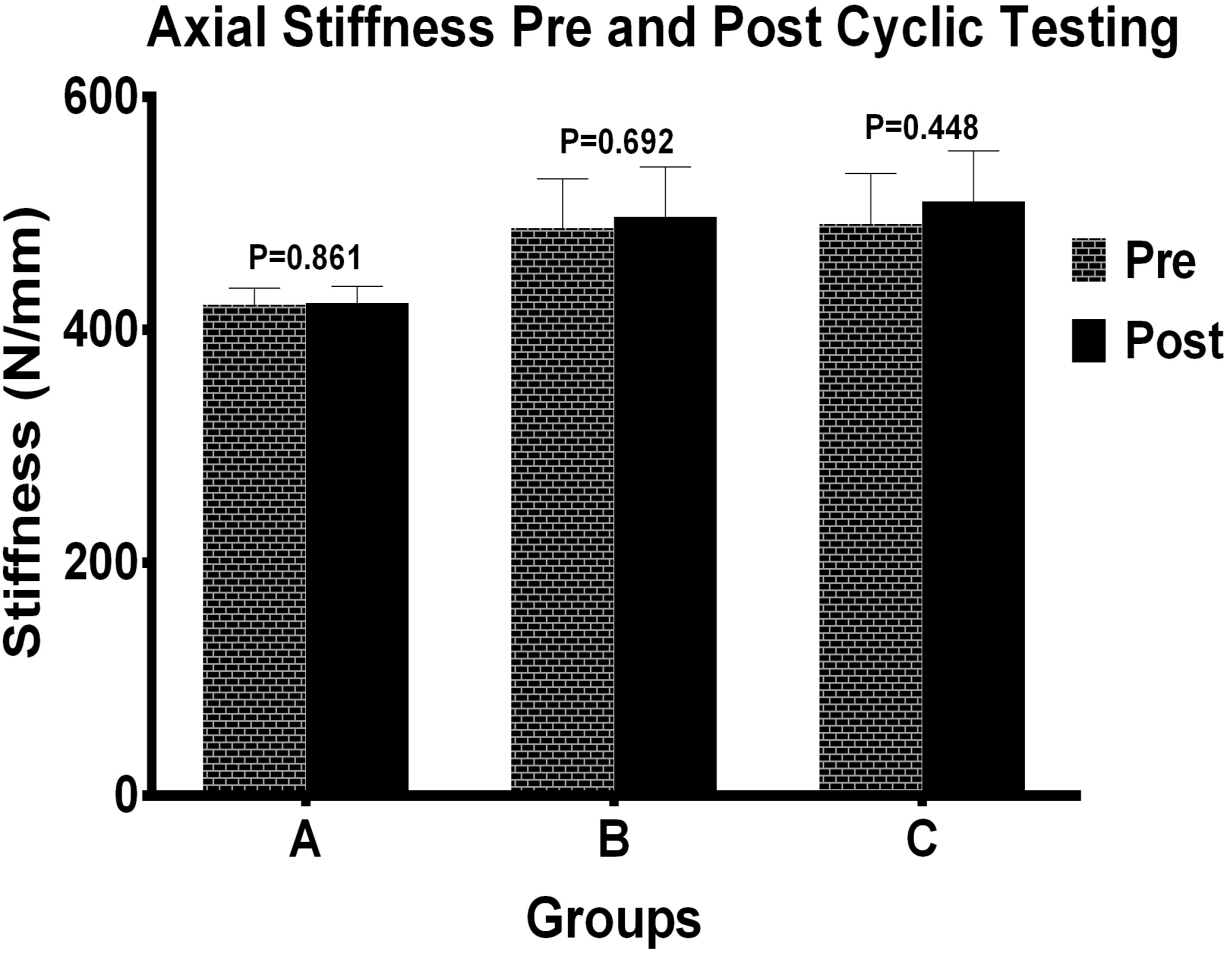
Axial stiffness before and after cyclic testing by different fixation groups. Mean stiffness of each group under axial loading condition were not significantly different (P>0.05). Numbers over bar indicated p values which were conducted through Independent-Samples T Test.

**Figure 4:**
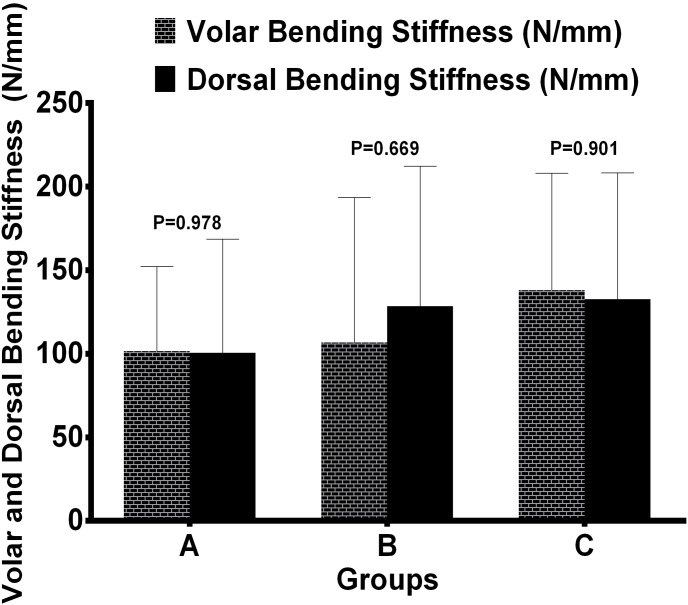
Mean stiffness results under volar and dorsal bending between groups. Stiffness values of each group under volar and dorsal bending load were not significantly different (P>0.05). P values were determined by way of independent sample test.

There was no statistically significant difference in stiffness among the 3 groups under axial compression before and after cyclic testing (Fig. 3). Final mean stiffness under dorsal bending in group A was 100.66±67.91 N/mm, and under volar bending 101.62±50.58 N/mm. In group B the mean stiffness under dorsal bending was 128.41±83.75 N/mm, and under volar bending 106.74±86.79 N/mm; and in group C it was under dorsal bending 132.74±75.54 N/mm, and under volar bending 138.10±69.93 N/mm. Mean stiffness to dorsal and volar bending forces were statistically similar between different fixation constructions (Table 1) and each group (Fig. 4).

**Table 1:** Results of Stiffness Under Different Conditions after Cyclic Testing.

Groups Compared	p Values for Axial Load	p Values for Volar Bending Load	p Values for Dorsal Bending Load
A versus B	.017	.902	.537
A versus C	.009	.385	.476
B versus C	.930	.454	.923

P<0.05 was considered to significant.

**Figure 5:**
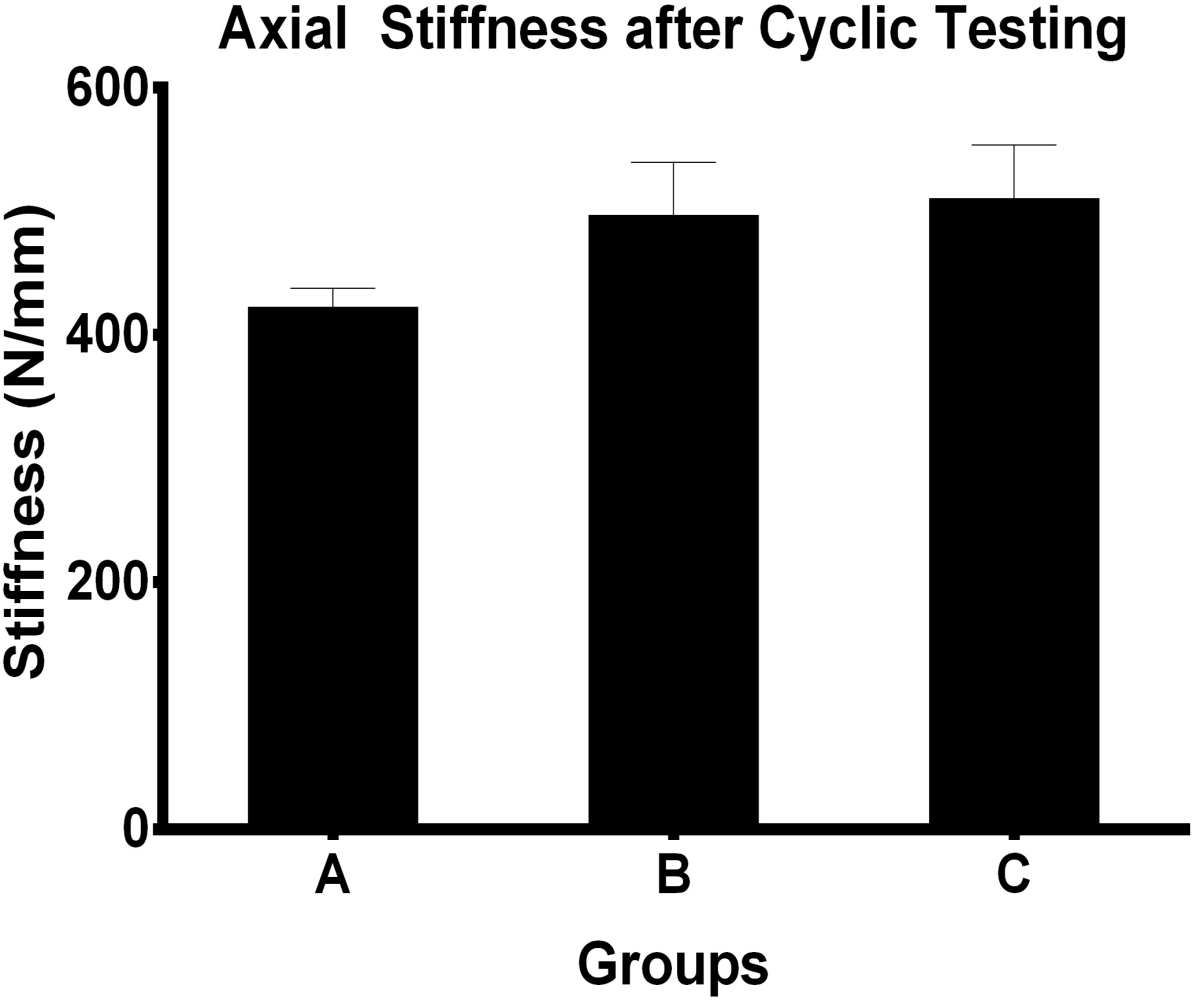
Comparison of postcycling axial stiffness among different groups. The stiffness were significantly different in all groups (P = 0.002) and P values were determined using One-way analysis of variance. While mean axial stiffness in group B and group C was statistically similar (*P* = 0.93).

Final mean stiffness in axial loading for group A, group B, and group C was 423.01±14.64 N/mm, 497.42±42.51 N/mm, and 510.69±43.25 N/mm, respectively and there was statistically significant difference in all three groups (P = 0.002) as we can see from figure 5. The stiffness of group A decreased obviously and differences are statistically significant with group B and group C (P = 0.017, P = 0.009). However, mean axial stiffness in group B and group C was statistically similar (P = 0.93).

**Figure 6:**
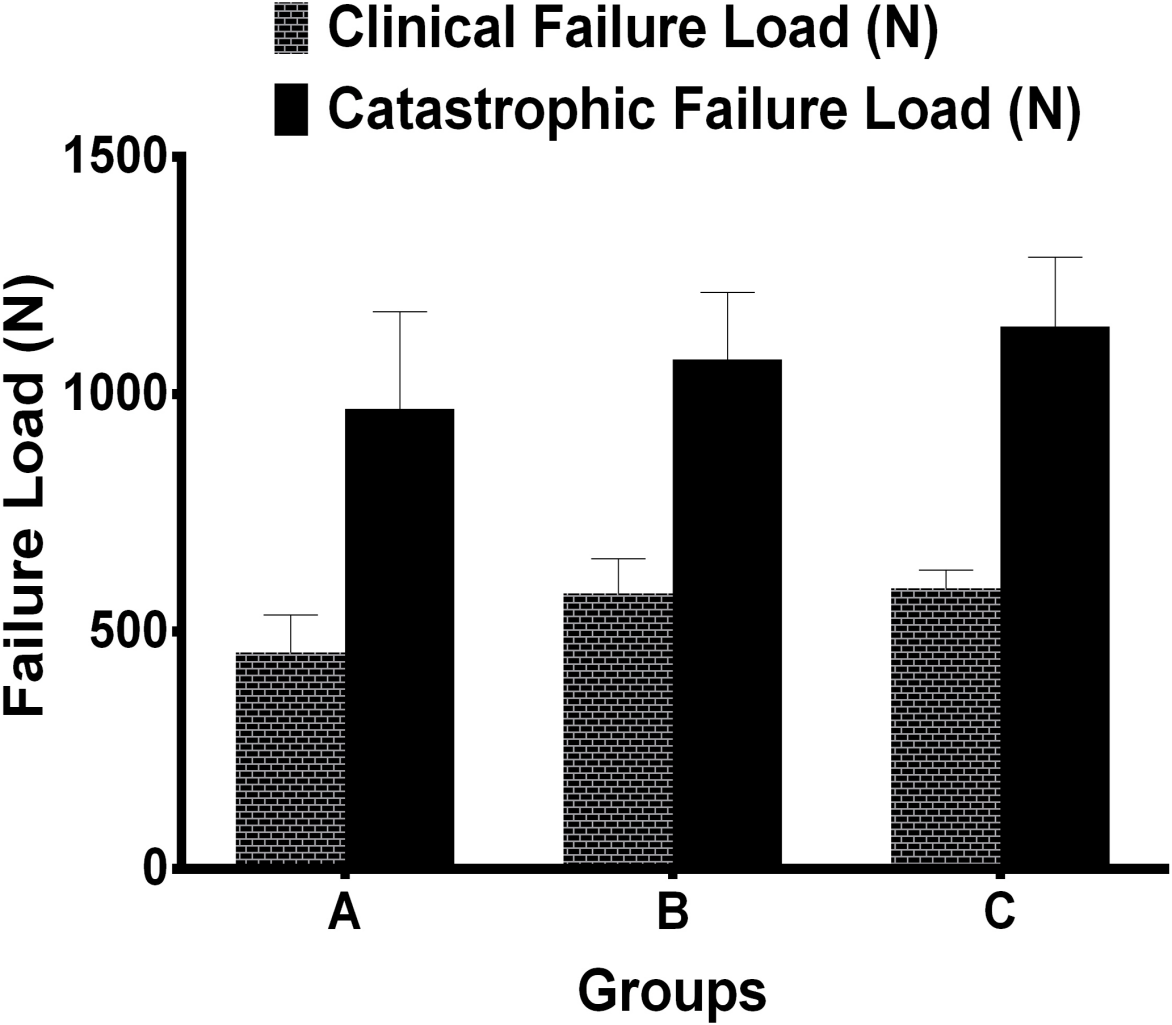
Comparison of mean load to clinical and catastrophic failure after cyclic conditioning by different fixation groups. As for load to clinical failure, there were statistically significant differences among all groups (P = 0.013), while the differences were not statistically significant for load to catastrophic failure (P = 0.483).

**Figure 7:**
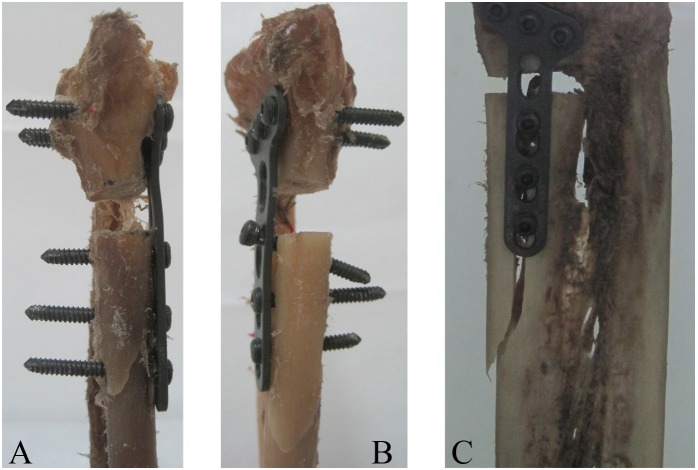
Catastrophic failure modes seen in specimens after failure testing: (A) A specimen failed by collapse of the location of distal radius diaphysis and slight deformation of mid-plate, (B) A specimen failed by collapse of the location of distal radius diaphysis and uncoupling of the proximal locking screw from the screw hole, (C) A specimen failed by collapse of the location of distal radius diaphysis.

As for the axial loads to clinical failure, the mean values for group A, group B, and group C was 456.54±78.59 N, 580.24±73.85 N, and 591.07±38.40 N. There were statistically significant differences among all groups (P = 0.013) except between pair group B and C (P = 0.80) as we can see from figure 6, and a gradual decrease in axial load to clinical failure was observed when shortening screws to 50% of the distance to the dorsal cortex, with mean values for Group A are 27% and 29% less than those for Group B and C, respectively. As for the axial loads to catastrophic failure, the mean values for group A, group B, and group C were 969.33±205.01 N, 1074±141 N, 1142.67±145.91 N, and the differences were not statistically significant among all groups (Fig. 6). However, mean values for Group A were 18% less than means for Groups C. Phenomena of clinical and catastrophic failure were observed and recorded during the tests. In all specimens, clinical failure with 2-mm displacement occurred without fixation destruction. Catastrophic failure occurred via fractures of different locations of the bone close to the volar plate, uncoupling of the locking screw from the screw hole and slight deformation of the plate (Fig. 7).

## Discussion

The dorsally angulated distal radius fracture is a common fracture that typically results from a fall with the forearm in pronation and the wrist in extension. The treatment goal should be anatomic reduction, stable fixation, and early mobilization of the wrist and forearm to avoid the potential disability caused by a malunion and/or prolonged immobilization. Obtaining and maintaining an anatomic reduction until the fracture is healed has resulted in a good clinical outcome. For this reason open reduction and internal fixation has become an increasingly common treatment for these injuries. Good outcomes have been reported with dorsal plates that can buttress the comminution of the dorsal cortex and maintain reduction of the dorsal displacement of the distal fragment [22], [23], [24]. It can be technically demanding, however, to place certain plates on the dorsal surface of the distal radius because of the irregularity of the distal dorsal radius. In addition there is limited soft tissue between the skin and bone surface, which can result in symptomatic hardware prominence. Besides, there also can be extensor tendon irritation or rupture from direct contact with a prominent dorsal plate or screws [25], [26], [27]. Thus in an attempt to avoid tendon irritation, it is suggested that dorsal plates should be low profile.

Volar locking plates have been designed to reduce extensor tendon complications observed with dorsal plates while still delivering stable fixation because volar plates with locking distal components can avoid the impaction of the cancellous bone in the distal fragment resulting in poor screw purchase and offer superior rigidity and axial loading strength, especially in the elderly [28], [29]. A volar plate has less potential than a dorsal plate to cause tendon complications in part because of the ability to cover the plate with the pronator quadratus (PQ) muscle, thereby avoiding direct contact of the plate with the flexor tendons. Though pronator quadratus muscle is incised along distal radius border during surgery, the repaired PQ has little effect on the stability of whole fixation system and functional recovery [30], [31], [32], [33]. However, with the increased use of volar locking plates, the tendon complications such as extensor tenosynovitis, extensor pollicis longus rupture have become more common owing to that fact that dorsally prominent screws and flexor tendon synovitis occurs occasionally when the volar plate is placed distal to the “watershed line” and prevents protection of flexor tendons by the pronator quadratus muscle. So we investigate whether it is necessary to use distal bicortical fixation and place the extensor tendons at potential risk, or whether unicortical distal fixation is sufficient to provide early stability. The purpose of the present study was to compare the biomechanical properties of distal locking screws with different length in an extra-articular osteoporotic distal radius fracture model.

In this study, we removed a section of metadiaphyseal bone to simulate a comminuted distal radius fracture. Because the model was designed to study the effect of different locking screw lengths in the distal fragment in one volar plate system, we deliberately limited the fracture variables to a simple model to examine specific stability in fixation with different locking screw lengths. Contrary to earlier published studies with synthetic radial bone [24], [34], [35], [36], we used true radial and ulnar bone with interosseous membrane and distal radioulnar ligaments undisturbed. Although a sawbone model can ensure consistency between specimens and obviate the need for computed tomography to assess for bone quality or substantial structural differences between specimens, these constructs cannot represent their in vivo states more closely.

The loads used in this study were designed comparable to the loads applied to the distal radius during early postoperative hand and wrist mobilization. Loads transmitted to the distal radius are about 50 N for each 10 N of grip forces with varies hand positions and radius lengths [37]. Although the forces of compression on the distal radius in vivo have not yet been clearly defined, several studies have suggested that compressive forces created by light active motion of the wrist do not exceed 100 N. Combined forces of motion of the wrist and the digits do not exceed more than 250 N [38], [39], [40], [41]. So in the present study, the specimens were performed under load control to a force of 150 N for axial compression and a force of 50 N for volar and dorsal bending. Axial and bending stiffness before and after cyclic loading, load to failure of specimens with different lengths of fixation, has been measured in cadaveric models. Because the loads close to the fracture area are always changing and repeating when active mobilizations of the fingers are performed during the acute healing period, cyclic loading rather than single loading was included in the experiment, since it better simulates physiological loading. Matched pairs and equal numbers of right and left extremities were tested to minimize anatomic and bone quality differences as a confounding factor.

In order to determine the effect of fixation with different locking screw lengths, we compared the mechanical performance of 50%, 75% length and bicortical screws fixations. In the osteoporotic distal radius fracture model, it was demonstrated that 75% length unicortical distal locked fixation produces a construct that was as stiff as fixation with bicortical screws. After cyclic testing that simulated fatigue scenario during the initial healing period, both types of fixation had similar mean stiffness and load to clinical and catastrophic failure were not significantly different, while a gradual decrease in axial load to clinical failure was observed when shortening screws to 50% of the distance to the dorsal cortex. The load to clinical failure forces obtained in our study ranged from 323.83 to 673.74 N among all groups, so it was proved that all construct fixations would bear light active motion during early period of postoperation. We believe that the mechanical equivalence (clinically and statistically) of unicortical and bicortical distal fixation should be viewed as evidence in favor of placing unicortical fixation in clinical practice. Unicortical distal fixation of distal radius fractures is potentially advantageous to bicortical fixation for some reasons. First, bicortical fixation places the extensor tendons at risk for irritation, synovitis, and rupture as a result of dorsally protruding screws. Second, extensor tendons can be injured with drill penetration during operation treating dorsally comminuted fracture and this inciting injury may progress to tendinitis or tendon rupture.

Based on previous data analyses, we considered 6 specimens each experimental group to be sufficient to detect the difference in final axial stiffness between the constructs with 80% power. Although we planned and conducted this study with power analyses consistent with prior investigations, several analyses were underpowered. Post-hoc analysis demonstrated appropriate power values when analyzing final axial stiffness and axial loading force to 2-mm displacement; however, we would have needed at least 19 specimens per group to detect whether the groups have difference as with final bending stiffness and axial loading to catastrophic failure. Even though, there is of less importance to detect the statistical significance of bending force condition because axial compression is just the main force transmitted to radius, as for axial loading to catastrophic failure, the result may be unreliable because of the poor bone quality of each group.

It is difficult to assess the penetration of bicortical screw tip dorsally during fracture fixation with standard lateral view. Because the dorsal surface of the distal radius has complex three-dimensional shape such as Lister tubercle which prevents recognize penetrating screws tips on a two-dimensional image. Several methods of radiological examination have been proposed such as oblique lateral view in pronation and supination and dorsally tangential view of distal radius [42], [43], [44]. However, both approaches have limitations to a certain degrees. On the other hand, distal comminuted fragments make it difficult or impossible to select appropriate screw lengths using a depth gauge. So distal bicortical fixation adopted in clinical practice in the metaphyseal and epiphyseal areas of the distal radius is at risk of screw prominence resulting in extensor tendon irritation.

In our study, almost all the constructs failed during catastrophic testing by collapse of the location of distal radius diaphysis or uncoupling of the locking screw from the screw hole. These kinds of failure have been observed after mechanical testing of cadavers with fractures of the distal radius in other published studies [45], [46]. The outcome was directly related to the biological properties of the bone with osteoporosis, which can be dramatically improved via increases in sclerotin. A similar bone with more bone substance will resist collapsing failure.

This study has some limitations that are inherent with using a cadaver model for simulating the in vivo scenario. These shortcomings involve the usual concerns associated with a cadaveric study such as the limited number of specimens (open to type II error), the variability in specimen age, and bone mineral density (BMD). Secondly, the muscles and tendons were stripped from the specimens in the study and any dynamic stability that would normally be provided by the musculotendinous tissues was not present. Thirdly, although dorsal and volar bending was tested as well as axial compression, the in vivo loading will be more complex and a torsion component may be included. These rotational forces through the radius are commonly experienced during pronation–supination of the forearm, which are important components in most postoperative rehabilitation protocols. Finally, the fracture model in our research was limited to an extra-articular fracture in which no coronal and sagittal plane fracture, and cannot entirely represent various types of comminuted fractures of osteoporotic distal radius found in practice.

## Conclusions

In summary, our study demonstrated that distal unicortical fixation with appropriate length screws provides adequate stiffness and strength for the internal fixation of extra-articular distal radius fractures during the acute healing period (6 weeks). So there is a thimbleful of effect on construct strength by decreasing screw length. We recommend at least 75% length unicortical screw fixation to minimize the risk of over-projection resulting in extensor tendon complications and suggested active range of motion should be instituted during the early period of postoperation to avoid wrist joint stiffness or disuse atrophy.
